# AI-Generated Scientific Papers: Crisis? What Crisis?

**DOI:** 10.1523/ENEURO.0470-25.2025

**Published:** 2026-01-07

**Authors:** Christophe Bernard

Picture a man in a deckchair, umbrella overhead, relaxing with a drink in hand—while surrounded by industrial wasteland and decay. This was the iconic 1975 album cover for Supertramp's *Crisis? What Crisis?* The image perfectly captured the cognitive dissonance of denying catastrophe while sitting in its midst. Rick Davies conceived the artwork to satirize how some responded to England's economic crisis of the mid-1970s: “Crisis? What crisis?”

Fifty years later, I find myself in my own version of that deckchair—though instead of industrial ruins, I am surrounded by what may be “arguably the largest science crisis of all time.” And just like that man with his parasol, I am tempted to pretend everything is fine (Figure 1).

But it is not fine. Not even close. We are facing an uncomfortable truth: the scientific literature is being flooded with fraudulent papers on an industrial scale. This crisis threatens to erode public trust in research at the very moment we need that trust most.

## The Paper Mill Industrial Complex

Paper mills are commercial operations that mass-produce research articles for paying “authors”—scientists under career pressure to publish or perish. These companies have industrialized the research process: they harvest public databases, apply standardized analytical pipelines, generate AI-written introductions and discussions, create publication-ready figures, and sell complete manuscripts with guaranteed authorship slots.

The scale is staggering. Recent estimates suggest hundreds of thousands of fake publications are produced each year, with the number accelerating rapidly thanks to generative AI. This represents what some have called “arguably the largest science crisis of all time”—a crisis that threatens to erode public trust in research at the very moment we need that trust most.

And here is the uncomfortable reality: we, the scientific community, are funding this fraud. Public money—taxpayer dollars, euros, yuan, and roubles—pays for these fabricated publications through institutional budgets, grant funds, and publication fees. Meanwhile, the citizens who fund our work are losing faith in the very enterprise they support.

## Why This Matters Beyond Academic Integrity

The consequences go far beyond individual careers or journal reputations:

- Editor and reviewer fatigue: We waste countless volunteer hours evaluating sophisticated frauds.
- Irreproducible experiments: Other scientists waste time and resources trying to build on fabricated work.
- Misguided research directions: False findings steer entire fields down dead ends.
- Medical harm: In biomedicine, fraudulent data can influence clinical practice. See, for example, this recently retracted paper, just look at their Figure 1. This is bad as the paper was claiming autism diagnosis.
- Erosion of public trust: When fraud is exposed—and it increasingly is—society's confidence in all science diminishes (cf. the autism paper cited above). The public funds our research through their taxes, their donations to medical charities, and their participation as research subjects. They trust us to be honest recipients of that investment.

**Figure 1.**
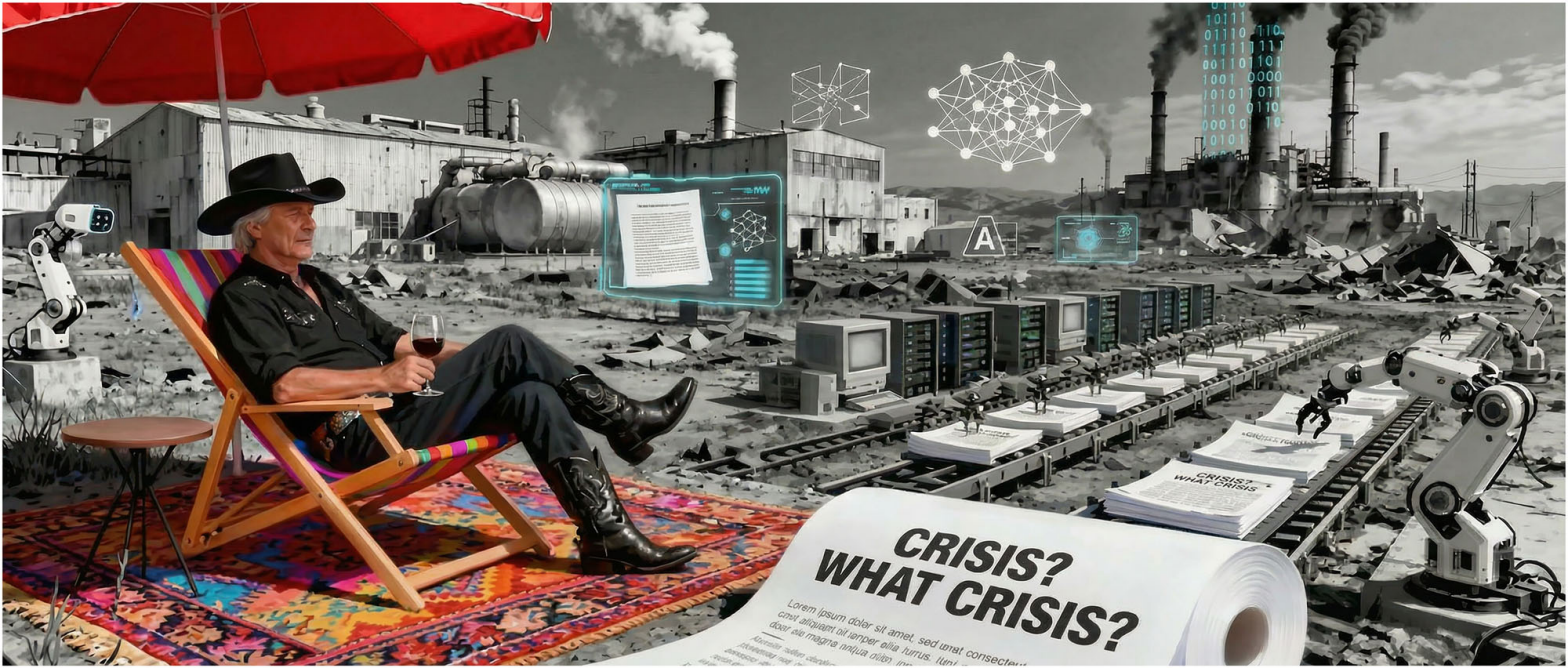
Perplexity-generated image with instructions: ditor_in_black_stetson_and_black_cowboy_boots_relaxing_while_AI_paper_mills_ massproduce_fake_science__CRISIS__WHAT_CRISIS___pays_homage_to_Supertramp's_iconic_album.png.

That last point is what truly haunts me. When we tolerate industrial-scale fraud, we betray the public's trust.

## The Root Problem: Publish or Perish

This crisis did not emerge randomly. It is a direct consequence of how we evaluate and reward scientific work. When hiring committees prioritize publication counts over research quality, when funders use impact factors as proxies for merit, when institutions measure prestige by citation metrics, we create powerful incentives for gaming the system. Paper mills exist because academia incentivizes publication in ways that make fraud economically rational.

The problem is evolving rapidly. Generative AI is making fraudulent publication faster, cheaper, and harder to detect. Our best screening tools—the industry standard updated constantly with new markers—struggle to keep pace. A low-effort paper that uses automated pipelines to analyze public data and generate text may be flagged for some AI use, but distinguishing fraud from legitimate (if uninspired) research becomes increasingly difficult.

## The Philosophical Question: What Is Science?

If a paper mill uses automated pipelines to mine public databases and the resulting analysis is statistically valid, is that science? I would argue: No. And Claude Bernard would have been the first to raise a barricade.

Science is not merely the mechanical application of methods to data. Science is the human process of asking meaningful questions, designing experiments to test hypotheses, interpreting results in context, and contributing to our collective understanding. Science requires intellectual investment, curiosity, skepticism, and genuine engagement with ideas. Again reading the classics (a must do for all present and future scientists) provides the necessary perspective to understand the magnitude of the problem (Bernard, 1865).

A paper mill product—even one with valid results—lacks the essential ingredient: *thinking*. There is no scientist behind it who genuinely wondered about a phenomenon, who iterated on experimental design, who grappled with unexpected findings, and who placed results in the broader context of their field. There is only an algorithm optimized for publication, not for knowledge.

As you know, *eNeuro* values transparency. We have received papers where data mining is used to extract results and which appear AI or paper mill generated. What should we do with them? There is no correct answer to this question, as there is no absolute truth regarding what Science is or should be.

## What Needs to Change

I was honored to participate in shaping the Stockholm Declaration, a comprehensive action plan drafted at the Royal Swedish Academy of Sciences in June 2025 (Sabel and Larhammar, 2025) to address this crisis, which requires systemic reform across multiple levels:

1. *Academia resumes control*

We must shift from profit-driven commercial publishing to nonprofit, scholar-led models where academics control journals and authors retain copyright. *eNeuro*, as a journal owned by the Society for Neuroscience and committed to transparency, already embodies these principles.

2. *Reward quality, not quantity*

We must end the “publish or perish” culture that incentivizes gaming the system. Hiring, promotion, and funding decisions should evaluate research on its merits—the depth of thinking, the rigor of methods, the significance of findings—not on publication counts, impact factors, or *h*-indices. This is not merely about fairness; it is about redirecting the incentive structure that makes fraud profitable.

3. *Develop independent fraud detection*

We need researcher-controlled, nonprofit organizations (not publishers with conflicts of interest) to develop automated systems for detecting fake publications, to maintain registries of fraudulent papers and paper mills, and to improve transparency throughout the publishing ecosystem.

4. *Implement legislation and policy*

Publishing fraud must have consequences. We need legal frameworks that define and sanction paper mill operations, that protect whistleblowers, that require transparency from publishers, and that hold institutions accountable when they artificially inflate publication numbers.

## What *eNeuro* Is Doing—and What You Can Do

At *eNeuro*, we have always prioritized transparency:

- Our double-blind review process reduces bias.
- Our consultation-consensus approach ensures reviewers and editors speak with one voice.
- Our review synthesis provides clear, factual feedback to authors.
- Our commitment to publishing negative results and registered reports contributes to fight publication bias.
- We have no “confidential comments to editors”—everything is transparent.

However, transparency alone cannot solve industrial-scale fraud. We need collective action.

## I Urge Every Reader to

1. “Advocate within your institution for quality-over-quantity evaluation metrics”
2. “Support researcher-led, nonprofit publishing” with your submissions and service
3. “Report suspected fraud”—be a scientific guardian, not a silent bystander
4. “Educate the next generation about integrity,” not just about productivity

## The Choice before Us

We are at an inflection point. The paper mill industry is growing. AI is making fraud cheaper and harder to detect. The incentives that drive scientists to purchase fake publications—career pressure, evaluation metrics, and institutional rankings—show no signs of diminishing.

However, we can choose what kind of scientific culture we create for the next generation.

*eNeuro* will continue to lead with transparency, to innovate our processes, to serve the neuroscience community rather than shareholder profits, but a journal cannot reform a system alone.

## This Is a Call to Action for All of Us

The public trusts us with their resources, their health, and their hope for progress. We owe them honest science—not just correct results but authentic inquiry conducted by real humans genuinely seeking to understand the brain.

The roots of this crisis are embedded in how we structure academic careers and evaluate scientific merit. Solving it requires all of us—funders, institutions, publishers, legislators, and scientists—to unite around integrity and to reconstruct incentive systems that reward genuine discovery over publication volume.

Now we need the courage to make that change. I hope you will join me.

*Crisis, what crisis?* This one.

*Note: I participated as an individual expert in the June 2025 Stockholm conference and cosigned the Declaration. Views expressed are my own and represent my perspective as Editor-in-Chief of eNeuro. It does not engage the Society for Neuroscience.*

Christophe Bernard

Editor-in-Chief, *eNeuro*

Director of Research Exceptional Class, INSERM, Institut de Neurosciences des Systèmes, Marseille, France
